# Results after multivisceral resections of locally advanced colorectal cancers: an analysis on clinical and pathological t4 tumors

**DOI:** 10.1186/1477-7819-10-39

**Published:** 2012-02-15

**Authors:** Cem Gezen, Metin Kement, Yunus E Altuntas, Nuri Okkabaz, Mesut Seker, Selahattin Vural, Mahmut Gumus, Mustafa Oncel

**Affiliations:** 1 Department of General Surgery, Kartal Education and Research Hospital, Istanbul, Turkey; 2 Department of Medical Oncology, Kartal Education and Research Hospital, Istanbul, Turkey; 3 Medical College of Gumushane University, Gumushane, Turkey

**Keywords:** Colorectal cancer, multivisceral resection, locally advanced colorectal cancer, pT4 tumor, morbidity and mortality, survival

## Abstract

**Background:**

Locally advanced colorectal cancers are best treated with multivisceral resections. The aim of this study is to evaluate early and late results after multivisceral resections.

**Methods:**

All patients operated for primary colorectal cancer between 2001 and 2010 were -reviewed. These were compared within the patients underwent single organ and multivisceral resections: demographics, tumor and procedure related parameters, perioperative results, early oncological outcomes and 5-year survival.

**Results:**

A total of 354 patients (59.6 ± 13.8 years old, 210 [59.3%] males) were abstracted. Ninety (25.4%) patients underwent multivisceral resections for clinical T4 tumors and en-bloc R0 resection was achieved in 82 (91.1%). Only 31 (34.4% and 8.8% of clinical T4 and all cancers, respectively) cases had actual adjacent organ invasions (pT4). Males (20%) had lower risk for locally advanced tumors than females (33.3%) (p < 0.05). PT4 cancers were more common, if the clinical T4 tumor is located in the colon (48.8% vs 21.3%; p < 0.01). Laparoscopy was seldom initiated and the risk of conversion was higher in clinical T4 tumors (p < 0.05). The rates of sphincter-saving procedures were not different. Operation time, bleeding and transfusion requirements increased when multivisceral resections were necessitated (p < 0.05), but hospital stay, complications and 30-day mortality rates were similar. The 5-year survival rates were identical (p > 0.05).

**Conclusions:**

Clinical T4 tumors are not rare and more common in women. An actual invasion (pT4) may be observed in one third of all clinical T4 tumors, and more frequent in colon cancers. An en-bloc, R0, multivisceral resection may be achieved in most cases. Multivisceral resections do not alter the rates of sphincter-saving procedures, morbidity and 30-day mortality; do not worsen survival but increase operation time, intraoperative bleeding and perioperative transfusion requirements.

## Background

Colorectal cancer is a common problem in western countries regarding incidence and death rates [1]. It is also estimated to be increasing in developing countries. A recent analysis by Ministry of Health has shown that the age standardized rates for colorectal cancer are 12.1 and 8.0 for men and women, making it the third and the second most common tumor in men and women, respectively, in Turkey [2]. If the cancer has grown through the wall of the colon or rectum and into other adjacent tissues or organs, it is named as a T4 or a locally advanced tumor which is detected during 5 to 22% of all surgical interventions performed for the management of colorectal cancer [3,4].

Surgery remains the principal treatment technique in most of the patients with colorectal cancer, even for the most of those with a locally advanced tumor. The decision-making for a surgeon confronting a T4 colorectal cancer is somehow challenging, since the adhesions between the cancer and the adjacent tissue have an unacceptably high risk of being malignant and the intraoperative assessment of nature of adhesions are often inaccurate [5]. Accordingly, guidelines have recommended en-bloc multivisceral resections for treatment of these tumors, since most studies have shown that this procedure improves the possibility of R0 margins associated with a better local control and improved overall survival [6-8]. However, surgery related mortality rates after multivisceral resections have been reported to be up to 12% [9-11]. Since limited number of studies included comparative data and the results are conflicting, it remains unclear whether or not multivisceral resections are making worse the early postoperative non-oncological results compared to standard single organ removals. Thus, this study aims to evaluate the results after multivisceral resections performed for the treatment of locally advanced colorectal cancers, and compare the outcomes with those obtained from standard resections.

## Methods

Institutional review board of Kartal Education and Research Hospital approved the design and content of the study prior to data abstraction (Reference number: B104ISM4340029/1009/3). A chart review was initiated for all patients operated for colorectal cancer between January 2001 and July 2010 in our institution. In addition, missed or confirmative information was obtained from computer-based records that have been used to collect prospective data in our unit since 2006. In order to standardize the information, data about emergency procedures were excluded. Furthermore, patients undergoing operation for a benign or recurrent disease, those presenting with only dysplasia or in situ cancers or those with cancers other than adenocarcinomas were excluded from the study and further analyses. The study patients were routinely discussed in a multidisciplinary council prior to initiation of the treatment, if the preoperative evaluation tools showed a possible risk in achieving R0 resection, and the indication for surgery particularly in patients with a rectal cancer was decided only if an R0 resection was predicted. Advanced staged (T3-4 or node positive) tumors located at two thirds of the rectum received neoadjuvant chemoradiation therapy. Patients were routinely received a total colonoscopy, unless an obstruction was not the case. In addition to the abdominal ultrasonography, which was used in all cases as the initial diagnostic tool particularly for discovering hepatic metastasis; colon and rectal cancer patients were evaluated with computed tomography (CT) and magnetic resonance imaging, respectively. Thorax was examined with either a plain graph or a CT which was the case in recent years. Patients with suspected clinical T4 tumors received further diagnostic tools including CT-angiograph, or other conventional imaging tools with barium meal when indicated by the council. All procedures were performed or supervised by a single surgeon (MO). During the initial stage of the study period, the operations were performed via a laparotomy; however since 2006, laparoscopy and hand-assisted laparoscopy have gradually become the preferred techniques for all colorectal tumors. Laparoscopic resection was sometimes achieved with 4 trocars, but generally a 5^th ^one was required, especially when the complete mobilization of the splenic flexure was necessitated. Laparoscopic-hand assisted operations were performed as described in our previous paper and were generally preferred for low rectal tumors treated with a sphincter-saving procedure [12].

Following aspects were investigated: demographics, tumor localization, invaded organs and actual pT4 rates, operative method (type and technique [laparoscopic, open, conversion] of the procedure, completeness of the resection [R0, R1 or R2] and the causes for R1-2 resections, presence or absence of en-bloc resection), incidence of preoperative distant metastasis and synchrone metastasectomy rates, operation time, intraoperative bleeding, the perioperative transfusion requirements, postoperative complications and 30-day mortality, length of stay, pathological data (type and differentiation of the tumor, number of harvested lymph nodes, status of surgical margins, presence/absence of vascular and perineural invasion of the tumor, T stage and node status), follow-up periods and survival.

Complete removal of the tumor without any microscopic (R1) or macroscopic (R2) residue was defined as R0 resection. If the mass was removed together with adjacent structures, but not separately, in case of a tumor adhering to the neighboring organs; the procedure was named as an en-bloc removal. Standard resection for a colorectal tumor without any additional organ removal was named as a single organ resection. If the tumor was adjacent to another organ (locally advanced or clinical T4 tumor), which necessitated the removal of multiple organs; the procedure was defined as a multivisceral resection. Only if pathological examination showed an actual invasion of the tumor to the adjacent organ in case of a multivisceral resection, it was considered as a pT4 cancer, otherwise the cancer was labeled as a false-T4 tumor. The perioperative and oncological data obtained from the patients who underwent multivisceral resections due to clinical T4 tumors were compared with those abstracted from single organ resections in order to find out the short- and long-term consequences of multivisceral resections. In addition, the demographics and tumor locations were also compared between pT4 and false-T4 tumors in order to find out possible factors that may play a role in actual pathological invasion to the adjacent organs.

### Statistical Analysis

Data were analyzed by using SPSS 15.0 for Windows. Results were given as percentages, mean and standard deviations or median and ranges. Quantitative and qualitative variables were compared with student's t-test and chi-square test, respectively. The survival rates were calculated with the Kaplan-Meier test and a log-rank was used to compare different survival curves. A p value less than 0.05 was considered significant.

## Results

A total of 408 patients (241[59.1%] males with a mean [± SD] age of 59.1 ± 14.2 years) underwent an elective procedure for malign or pre-malign colorectal tumors during the study period in our department. Among those, 54 (13.2%) cases were excluded since their pathological examinations revealed dysplasia or insitu cancer without an invasion (n = 14; 3.4%), tumors other than adenocarcinomas (n = 5; 1.2%) or because the operation was performed for a recurrent disease (n = 35; 8.6%); leaving 354 patients (59.6 ± 13.8 years old, 210 [59.3%] males) for the further analyses.

Among the study patients, 90 (25.4%) underwent a multivisceral resection for a locally advanced (clinical T4) tumor. An average number of 1.6 ± 0.9 (range: 1 to 4) additional organs were resected in these cases, and ovaries (n = 24), urinary bladder (n = 23) and small bowel (n = 19) were the most frequently removed ones. However, only 31 (34.4%) had real adjacent organ invasions and were classified in pT4 group. This pathological analysis revealed that the rate of pT4 tumors was 8.8% among all primary (n = 354) colorectal adenocarcinomas. Examinations showed that the absolute pathological T stages (n = 90) of false-T4 tumors (n = 59) were pT3 in 48 (53.3%), pT2 in 9 (10.0%) and pT1 in 1 (1.1%) cases; and no residual tumor was found in a patient's specimen (1.1%) who received neoadjuvant chemo-radiation therapy. Demographics were similar within the patients with pT4 (n = 31, 19 [61.2%] females, mean [SD] age was 61.0 ± 12.8) and false-T4 (n = 59, 29 [49.1%] females, mean [SD] age was 56.4 ± 15.6) tumors (p = 0.844 and p = 273 for age and gender, respectively). When only locally advanced tumors were considered, rectal cancers (10 out of 47; 21.3%) had a lower risk for having a real pathological invasion (pT4) than those located in the colon (21 out of 43; 48.8%) (p = 0.006). The actual invasion rates were high after pancreatic (66.7%), gastric (50%) and uterine (41.2%) resections and low after the removal of urological organs (7.1% for ureter and 4.3% for urinary bladder) (Table 1). Among those who underwent multivisceral resection, en-bloc removals were achieved in 84 (93.3%). However, the adjacent organs were resected separately in 6 cases, due to the technical reasons, in whom the tumors had invaded ovary (n = 3), small bowel (n = 2) or ureter (n = 1). In addition, in 2 (2.2%) cases, who had bulky colonic and rectal cancers in multiviseral resection group, en bloc duodenum and urinary bladder removals were completed and R0 resections were intraoperatively anticipated, however the pathological examinations revealed positive surgical margins (R1 resection). Thus, an en-bloc R0 resection was achieved in 82 (91.1%) patients who underwent multivisceral resections for colorectal cancer.

**Table 1:** The Additionally Resected Organs: The Rate of Resection as a Part of Multivisceral Resection and The Possibility of an Actual Invasion

Organs	Clinical-T4 (%)	Actual Invasion (%)
Ovary	24 (26.6)	7/24 (29.2)
Urinary bladder	23 (25.5)	1/23 (4.3)
Bowel	19 (21.1)	7/19 (36.8)
Uterus	17 (18.9)	7/17 (41.2)
Ureter	14 (15.5)	1/14 (7.1)
Vagina	13 (14.4)	3/13 (23.1)
Abdominal wall	12 (13.3)	3/12 (25.0)
Appandix vermiformis	7 (7.7)	2/7 (28.6)
Duodenum	6 (6.7)	2/6 (33.3)
Stomach	4 (4.4)	2/4 (50.0)
Pancreas	3 (3.3)	2/3 (66.7)
Prostate	3 (3.3)	1/3 (33.3)
Spleen	2 (2.2)	0/2 (0)
Colon	1 (1.1)	0/1 (0)
Coccyx	1 (1.1)	0/1 (0)
Gall bladder	1 (1.1)	0/1 (0)
Total	90 (100)	31/90 (34.4)

The demographic information and the localization of the tumor were evaluated in order to find out possible factors that may play a role in actual pathological invasion to the adjacent organs. Although there were more men (n = 210; 59.3%) than women (n = 144; 40.7%) operated for colorectal cancer, males (42 out of 210; 20%) had lower risk to have a clinical T4 tumor than females (48 out of 144; 33.3%) (p < 0.05). The distribution of single organ and multivisceral resections were identical among the tumors located in the colon and rectum (p > 0.05).

The perioperative data concerning single organ and multivisceral resections were compared. The rate of neoadjuvant chemoradiation therapy was similar within the single organ resection (n = 106, 71.6%) and multivisceral resection (n = 34, 72.3%) groups (p = 0.924). The reasons for the omission of neaoadjuvant chemoradiation therapy were as follows: tumor location at the upper rectum (n = 10), preoperative understaging (n = 2) and surgeon preference in a case with synchronous resectable hepatic metastasis. Laparoscopy was seldom initiated and the risk of conversion was higher in patients with clinical-T4 tumors (p < 0.05 for both comparisons). The rates of extended procedures (extended right/left hemicolectomy or subtotal resection or total proctocolectomy) for colon cancer, were also identical in patients who underwent single organ (31 [19.5%] out of 116) and multivisceral (15 [34.9%] out of 43) resections (p = 0.468). Similarly, the possibility of a sphincter-saving procedures (low anterior/intersphincteric resections/total proctocolectomy with stapled or hand-sewn coloanal or ileo-anal anastomosis) in single organ resection (101 [68.2%] out of 148) and multiorgan resection (29 [61.7%] out of 47) groups were alike in patients with rectal cancer (p = 0.407). Among those who had low anterior resections, the rates of intersphincteric resections were identical in single organ (27 [26.7%] out of 101) and multivisceral (9 [31.0%] out of 29) resection groups (p = 0.951). Intra- and post-operative data including length of hospital stay, and complications were similar within the groups, except operation time, amount of bleeding and transfusion requirement, which were significantly higher in cases that underwent multivisceral resections (p < 0.05 for all comparisons). 30-day mortality was observed in 4.2% of all patients (n = 15), and the causes for death were pulmonary embolism (n = 5), cardiac insufficiency or poor general condition (n = 5), intra-abdominal sepsis (n = 3) and pulmonary infection/pneumonia (n = 2) (Table 2).

**Table 2:** Demographics, Tumor Localizations, Operation Technique, Intra- and Postoperative Parameters, Complications and Pathological Features in Patients Who Underwent Single Organ and Multivisceral Resections

	Single organ resections (n = 264)	Multivisceral resections (n = 90)	p
Demographics			
Age	59.7 ± 13.7	59.4 ± 13.9	0.871
Gender (males)	168 (63.3)	42 (46.7)	0.005
Localization (Colon/Rectum)	116 (48.5)/148 (51.5)	43 (56.7)/47 (43.3)	0.527
Preoperative metastasis	32 (12.1)	11 (12.2)	0.980
Synchrone metastasectomy	10 (3.8)	4 (4.4)	0.759

Operation technique			
Conventional/laparoscopic	110 (41.7)/154 (58.3)	54 (60.0)/36 (40.0)	0.003
Conversion (%)	25/154 (16.2)	15/36 (41.7)	0.001
Intra- and Postoperative Parameters			
Operation time (min)	(n = 252) 203.0 ± 53.4	(n = 83) 256.4 ± 77.0	0.0001
Intraoperative bleeding (ml)	(n = 213) 250 0[1-4000]	(n = 68) 450 [100-2700]	0.0001
Total transfusion			
Rate	119/256 (47.4)	61/88 (68.5)	0.0001
Amount	0 (0-12)	2 (0-26)	0.001
Synchrone metastasectomy	10 (3.8)	4 (4.4)	0.759
Length of hospital stay	8.6 ± 6.8	9.0 ± 4.9	0.652

Complications	(n = 264)	(n = 90)	
Surgical site infection	13 (4.9)	6/90 (6.6)	0.590
Wound infection	6 (2.3)	3 (3.3)	
Evisceration	5 (1.9)	2 (2.2)	
Intraabdominal abscess	2 (0.8)	1 (1.1)	
Fistula	16 (6.0)	5 (5.5)	0.831
Anastomotic	14 (5.3)	2 (2.2)	
Urinary	1 (0.4)	2 (2.2)	
Biliary	1 (0.4)	1 (1.1)	
Anastomotic leakage	14 (5.3)	2 (2.2)	0.378
Ileus	11 (4.2)	5 (5.5)	0.568
Nonsurgical	5 (1.9)	3 (3.3)	0.429
Intrabdominal hemorrhage	7 (2.7)	4 (4.4)	0.483
Urinary retantion/leakage	2 (0.8)	1 (1.1)	0.999
Missed bowel injury	1 (0.4)	1 (1.1)	0.449
Reoperation	5 (2.0)	2 (1.1)	0.999
Intrabdominal hemorrhage	2 (0.8)	1 (1.1)	
Anastomotic leakage	2 (0.8)	0 (0)	
Missed bowel injury	1 (0.4)	1 (1.1)	
Overall	55 (20.9)	22 (24.4)	0.516

30-day mortality	11 (4.2)	4 (4.4)	0.999

T Stage			0.0001
pT0*/pT1/pT2/pT3/pT4	16 (6.1)/12 (4.5)/37 (14)/199 (75.4)/0	1 (1.1)/1 (1.1)/9 (10)/48 (53.3)/31 (34.4)	

Harvested lymph nodes	13.7 ± 7.3	16.0 ± 9.6	0.043

N Stage			0.227

pN (negative)/(positive)	148 (56.1)/116 (43.9)	54 (60)/44 (40)	

Vascular invasion	103 (39.0)	43 (47.7)	0.363
Perineural invasion	116 (43.9)	54 (60.0)	0.043

Differentiation			0.227
Poorly/Moderately/Well/Undetermined or missed	28 (10.6)/208 (78.8)/12 (4.5)/16 (6.1)	8 (8.9)/77 (85.6)/4 (4.4)/1 (1.1)	

Positive surgical margin (R1-R2 resection)	2 (0.8)	2 (2.2)	0.268

* includes only pT0 rectal tumors after neoadjuvant chemo-radiation therapy (complete response)

The oncological results revealed almost no significant difference within the groups for the most of the comparisons. As expected, the number of pT4 tumors was higher in multivisceral resection group (p < 0.01). But, after the exclusion of pT4 tumors, the distribution of different T stages among the groups was not statistically significant (p = 0.392). In addition, the amount of harvested lymph nodes and the rate of perineural invasion were less in single organ resection group (p < 0.05 for both comparisons) (Table 2).

**Figure 1:**
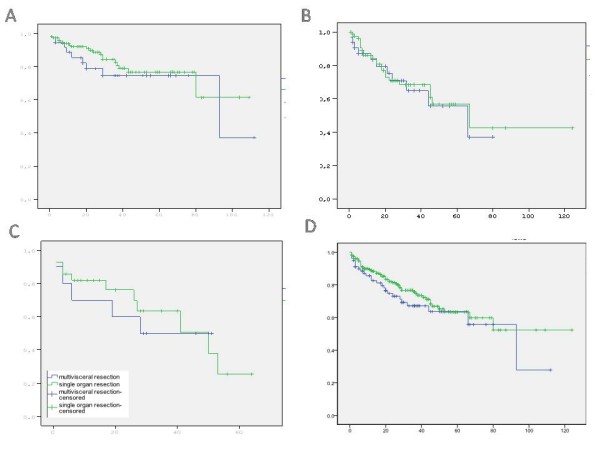
**Kaplan-Meier analyses revealed no statistical difference in overall survival between the groups when the comparisons included node negative (p = 0.374) (A), node positive (p = 0.765) (B) and metastatic (p = 0.788) (C) cases; as well as all patients (p = 0.397) (D)**.

A total of 45 (12.7%) patients (34 [12.9%] in single organ and 11 [12.2%] in multivisceral resection group) were lost in contact during the follow-up period. After mean follow-up periods of 22 (range 0 to 124) and 25 (range 0 to 76) months (p = 0.986), the 5-year survival rates were 64.6% and 69.4% for single organ and multivisceral resection groups, respectively; and Kaplan-Meier tests denied to reveal a significant difference in subgroup analyses regarding node negative, node positive and metastatic groups of patients (Figure 1).

## Discussion

Colorectal cancer is one of the most common malignancies observed, and the treatment generally includes a surgical intervention. However, the operation may sometimes be challenging if the tumor locally invades other intraabdominal organs which is the case in 5 to 20% of the patients. An en-bloc resection of the tumor with adjacent organs has been advocated in order to achieve an adequate surgical treatment in these cases. In many studies, the risk and outcome after multivisceral resections for locally advanced tumors have been discussed and mostly it is believed that an en-bloc resection of the mass with the adjacent structures is the best surgical option for the treatment of clinical T4 cancers [3,9,10,13,14]. However data remains conflicting, since some other series revealed that multivisceral resections may be associated with a higher morbidity rate [15,16]. In addition, early postoperative mortality up to 12% has been reported [11]. Accordingly, a recent observational national study on the SEER Registry has declared that only 33.3% of the 8380 patients with locally advanced colorectal cancer were managed with multivisceral resection, and stated the necessity of further research on this particular subject [17,18]. Thus, current study aims to evaluate the outcomes after multivisceral resections and particularly evaluate the safety of this challenging procedure and long term results.

We have analyzed a total of 354 patients operated for primary colorectal cancers in our institution within a period of almost 10 years, and among those, 90 (25.4%) required a multivisceral resection due to a locally advanced disease. Ovaries, urinary bladder and small bowel were the most frequently resected additional organs. The rate of abdominal wall resection is less than those presented in the previous data, probably because we did not consider and document in operation notes simple peritoneal resections as 'tumor invasion to the abdominal wall' unless muscular structure was penetrated by the tumor [3,10]. This is probably one of the reasons of low pT4 tumor rate in our study, since it has been demonstrated in previous studies that adhesions between tumor and other organs harbor malignant cells approximately 40% of cases, and sometimes increases up to 72.5% [3,7,9,10,16]. In the current series, pathological analysis has revealed that only one third of all clinically T4 tumors were actual pT4 cancers. Although current study does not evaluate the preoperative prediction for T4 cancers, it may be concluded that most of the clinical T4 tumors are recognized at the time of surgery, as has mentioned in previous studies [10,16]. In addition, pathological analyses denied a pT4 cancer after most of urological organ resections including ureter or urinary bladder, however in our opinion, these results do not justify blunt dissection of the tumor in case of a clinical T4 cancer invading urological organs since it is unpredictable whether or not an actual invasion exists.

The rates of clinical T4 tumors and R0 resections seem to be higher than previously published data [10,15-17]. The higher risk of confronting a T4 tumor in the current data may be speculated to be related to two factors: First, the early detection of colorectal tumors is rare in our district and most of the patients (93.2%) presented with pT3 or pT4 cancers as reported in our previous paper [19]. Secondly, since our unit takes part in a level 3 center, patients with more advanced tumors might be referred to our institution. Some may also question the indication for multivisceral resection in our series, particularly for those located in the rectum, since the actual invasion rate in urinary bladder was only 4.3% and the prostate resections were necessitated in only 3 cases, which might be lower than had expected. We do not know whether these findings were related to our strategy neoadjuvant chemoradiation application in all distally located advanced rectal cancers, or to the patient selection, which was performed by a multidisciplinary council that rule out to operate the patients who had a potential risk for R1-2 resections. In our opinion, this highly conservative strategy lead that a R0 and en-bloc removal was achieved in most (91.1%) of the patients who underwent a multivisceral resection.

Current study has investigated the patient and procedure related information of single organ and multivisceral resections. It was observed that females had a higher risk to undergo a multivisceral resection due to a clinical T4 tumor than males, and colon tumors carried a greater risk of a pathological invasion (pT4). This demographic difference was previously mentioned by others, but has not been statistically confirmed in a single institution study [10,20,21]. The data analyzing SEER Registry have also verified this finding and have concluded that females are under a higher risk for a multivisceral resection in both colon (odds ratio 1.45; range 1.29-1.63) and rectal (odds ratio 4.07; range 3.13-5.29) cancers [17]. It is hard to speculate why women with colorectal cancers are under a higher risk for clinical T4 and consequent multivisceral resections, but it may be related to the anatomic location of uterus and ovaria in women. It has been speculated that the female internal genitalia may cause a barrier effect against tumor invasion keeping urinary bladder safe which is a common location for tumor invasion in most series and it is difficult to remove. This anatomic construction in women may lead a higher en-bloc resection rate of genital organs that can be easily resected with the adjacent structures [5].

Current data revealed that more extensive lymphatic resections are performed and thus the number of harvested lymph nodes is increasing in cases, who underwent a multivisceral resection. On the other hand, current study has shown the number of extended colonic resections is elevated but not significantly higher than single organ resections, and the possibility of having a sphincter saving procedure has not changed. Furthermore, operation time, intraoperative bleeding, and the rate and amount of perioperative transfusions were significantly higher in patients who underwent a multivisceral resection, which was also the case in previous studies and was speculated to be related to the extent of the surgery [10,15,16]. The introduction of laparoscopy and the possibility of procedure completion without a conversion were also significantly less in patients with clinical T4 tumors. Besides, a laparoscopic multivisceral resection was achieved in 21 patients, which may demonstrate that laparoscopy may be used in selected clinical T4 colorectal cancers. In our opinion, locally advanced colorectal cancers and consequent multivisceral resections are challenging procedures that may alter surgeons' routine practice to a more aggressive procedure that may cause some adverse outcomes including an extended operation time, increased bleeding and transfusion rate and limitation in initiation and completion of laparoscopic technique; but they do not increase the possibility of abdominoperineal resections in case of rectal cancers.

There has been a disagreement in the literature on whether or not a multivisceral resection is associated with a higher complication rate. Most of the studies reported reasonable complication rates comparable to single organ resections [3,15,22,23]. Besides, others stated higher risks of morbidity up to 33 and 50% [10,24]. A comparative study evaluating short-term and long-term results after standard operations and multivisceral resections for colorectal cancer exposed the overall complication rates as 17.8% and 49.1% after these procedures, respectively (p < 0.0001). In this series, it was mentioned that higher morbidity rate might be related to the increased number of anastomosis and the extended resections [16]. Although some comparative studies denied an increase risk in postoperative mortality compared to single organ removals, others reported unacceptably high rates in patients underwent multivisceral resections [3,10,11,16,22,25,26]. Current study has revealed that patients who underwent multivisceral resections due to locally advanced tumors are not under a higher risk than those managed with single organ removals regarding the rates of postoperative complications (20.9% vs 24.4%) or 30-day mortality (4.2% vs 4.4%). Although some have reported excellent surgical complication risks as low as 1.4%, ours may be considered to be lower than expected [3]. It is probably because we had evaluated all patients in a multidisciplinary council prior to the operations, or may be related to the fact that the rate of multivisceral resections was more in patients with tumors located at the colon, which are obviously under a lower risk for complications compared to rectal cancers. In previous analyses, mortality rates between 0 to 12% were reported in patients underwent multivisceral resections [9-11,16]. We believe that the mortality rate in the multiple organ resection group in our study was acceptable and comparable to previously published data [9-11,16]. In contrast, the mortality rate in single organ resection group in our study seems to be higher than those reported in the literature, although some others have stated higher incidences as ours [14,26,27]. For example, in a national-based study in a French population, Mitry et al has reported a mortality risk of 6.2% in patients underwent a resection for cure [26]. Thus, in our opinion our data support that multivisceral resections can be performed with acceptable postoperative morbidity and mortality rates in patients with clinical T4 colorectal cancers as mentioned in previous studies [10,14].

The oncological outcome after multivisceral resection has the paramount significance. Previous data presented discouraging 5-year survival rates as low as 30 to 38% after multivisceral resections even in cases in whom R0 resection was achieved [10,22]. More recent studies have mentioned reasonable 5-year survival rates up to 76% in patients with negative lymph nodes after this procedure, but the results were not compared with those obtained from single organ resections [3,10,11]. In addition, it has been also shown that pT3 and pT4 tumors may have similar oncological results [15,16]. However, in our opinion, a comparison of oncological outcomes obtained from pT3 and pT4 tumors may include some bias and can not rationalize the necessity of multivisceral resections, since the final pathological examinations of clinical T4 tumors may present pT2 or pT1 cancers as observed in the current study (n = 9 [10%] for pT2 and n = 1 [1.1%] for pT1). Thus, it may be logical to compare the results of multivisceral and single organ resections in order to find out the realistic benefit in oncological perspective after this challenging procedure. Montesani et al gave their experience on 525 patients comparing the outcomes after single organ or multivisceral resections, and showed no difference in long-term results between the groups; however the 5-year survival rates were as limited as 30% in this series [22]. In addition to the finding revealing similar long term oncological results in single organ and multiple organ resection groups in the current study, subgroup analyses also have shown identical 5-year survival rates when only node negative, node positive and metastatic patients were considered. In our opinion, these consequences confirm that multivisceral resections may be warranted for all clinical T4 colorectal cancers in order to achieve acceptable long-term oncological benefit.

Current study may have some limitations predominantly causing from the retrospective design of the data abstraction and the missed information particularly belong to those operated during the initial phase of the study period. In addition, some data may interfere with other results, such as intraoperative bleeding and perioperative transfusion rates may be originated from the limited number of laparoscopic procedures in multivisceral resections, since previous multicenter randomized studies have shown that open technique may increase the amount of intraoperative bleeding and the requirement of transfusion [27]. However, in our opinion, besides these limitations, current data still support the principle that an en-bloc multivisceral resection should be decided in case of a clinical T4 tumor, since it may offer a reasonable survival rate with an acceptable increased risk in perioperative outcomes.

## Conclusion

In conclusion, clinical T4 tumors are not rare, and may be more frequently observed in females. Ovary, urinary bladder and bowel are the most commonly affected organs. An en-bloc, R0, multivisceral resection may be achieved in most of the cases. A true invasion (pT4) may be observed approximately in one third of all clinical T4 tumors and is more common among colon cancers. The rate of sphincter-saving procedures was identical within both techniques, but laparoscopic procedures are less commonly aimed and completed in cases that required a multivisceral resection. When compared with single organ resections, this technique does not increase morbidity but operation time, intraoperative bleeding and perioperative transfusion rate and amount. Survival rates after single organ and multivisceral resections are identical.

## Competing interests

The authors declare that they have no competing interests.

## Authors' contributions

Data abstraction: CG, NO, MS. Manuscript writing: MO. Editing and reviewing: YEA, SV, MG. Statistics: MK, NO. All authors read and approved the final manuscript.
